# Ending preventable maternal and child deaths in western Nigeria: Do women utilize the life lines?

**DOI:** 10.1371/journal.pone.0176195

**Published:** 2017-05-18

**Authors:** Titilayo Olaitan, Ifeoma P. Okafor, Adebayo T. Onajole, Olayinka A. Abosede

**Affiliations:** Department of Community Health and Primary Care, College of Medicine, University of Lagos, Lagos, Nigeria; University College London, UNITED KINGDOM

## Abstract

**Introduction:**

Nigeria which constitutes just one percent of the world population, accounts for 13% of the world maternal and under-five mortality. Utilization of health care services has been an important determinant of maternal and child outcomes. The vast majority of maternal and child deaths could be prevented if women utilize the available life lines. The study objective was to determine utilization of maternal and child health care services among women of child bearing age in Western Nigeria.

**Methods:**

A community based, cross sectional study was done in Oshodi/Isolo Local Government Area among women of child bearing age (15-49 years) with at least one child under five years. Multistage sampling was used to select 371 respondents. Data was collected with a structured, pretested, interviewer administered questionnaire and analyzed with Epi info 3.5.1. Summary and inferential statistics were done. Level of significance was set at 5%(p<0.05).

**Results:**

Of the 371 respondents interviewed, the health facility was used for antenatal care (74.3% n = 276), delivery (59.9% n = 222), postnatal services (77.9% n = 289), family planning services (28.8% n = 107), immunization (95.1% n = 353), growth monitoring (77.4% n = 287), nutritional services (64.7% n = 240) and treatment of childhood illness (49.6% n = 184). Only 31.5% (n = 117) of the respondents practiced exclusive breastfeeding and 82% (n = 263) of the mothers used oral rehydration solution for diarrhoea management. Maternal education significantly influenced utilization. In addition maternal age, employment status, number of children, spouse employment and educational status played significant roles.

**Conclusion:**

Utilization of maternal and child health services among respondents was above national average but not optimal, especially family planning services, exclusive breastfeeding and curative services for children. Interventions that improve maternal educational status and wealth creation should be undertaken to achieve the SDGs.

## Introduction

Every day, approximately 830 women die from preventable causes related to pregnancy and childbirth[1]. For every woman who dies in childbirth; a lot more suffer injury, infection or disease. Globally an estimated 289, 000 maternal deaths and maternal mortality ratio (MMR) of 210 maternal deaths per 100, 000 live births occurred in 2013 [2]. Nigeria accounted for 13% of all maternal deaths in 2013 [3]. “Similarly, every single day, Nigeria loses about 2,300 under-five year olds and 145 women of childbearing age” [4]. This makes the country the second largest contributor to the under—five mortality rate and maternal mortality ratio in the world.

Maternal, newborn and child healthcare service utilization remain important indicators for monitoring the progress of maternal and child health outcomes. Antenatal Care (ANC), delivery at health facilities with skilled professionals and postnatal care (PNC) reinforce the timely management and treatment of complications to reduce maternal deaths[5–7]. Family planning is another important component of the Safe Motherhood Initiative to reduce maternal deaths in developing countries[8]. Also reduction in under five mortality can be obtained by increase in use of vitamin A, immunization, oral rehydration therapy, long lasting insecticidal nets (LLINs), exclusive breastfeeding and treatment of common childhood illness (malaria) [9]. These interventions can be regarded as life lines as they serve as means through which lives of mothers and children can be saved or preserved when utilized.

However, utilization of these services remain low in Sub-Saharan Africa (SSA) and Nigeria [10–14]. According to National Demographic and Health Survey (NDHS) 2013 report, 63% of women received antenatal care from a skilled provider, 38% had skilled delivery and only 10% were currently using modern family planning. The same report indicated that under five mortality rate was still high at 128 deaths per 1000 live births; only 25% of children age 12-25 months were fully immunized and only 17% exclusively breastfed. These figures were much lower for rural than urban areas [15]. Studies have also revealed a very low usage of post natal care services in the country [16–17]. Maternal complications and perinatal mortality are highly associated with under-utilization of antenatal and delivery care services. The provision of skilled care before, during, and after childbirth saves lives of women and it also increases the chances of having healthy infants [1],[18]. World Health Organization (WHO) analysis shows that if 90% of women received ANC, up to 14% or 160,000 more newborn lives could be saved in Africa [19]. Also an estimated 17.1 million children’s lives have been saved since 2000, largely due to increased vaccination coverage against highly contagious viral diseases [20].Over 800,000 children’s lives could be saved every year among under fives, if they were optimally breastfed[21]. Many more deaths could be prevented if all the maternal, newborn and child health (MNCH) care services were optimally utilized between 2016 and 2030, in order to meet the Sustainable Development Goal (SDG) 3 [22].

Despite the existence of national programs for improving MNCH in Nigeria, the mortality and morbidity rates are still high as the progress of decline is slow [23]. The benefits of health care seeking and positive health behaviours are related strongly in settings and subgroups where socio-economic and public health resources are constrained[24]. ^“^The utilization of health care services is a complex phenomenon related to the availability, quality, cost of services, social structure, health belief and characteristics of the users”[25–26]. The extent of use varies from one community to another and also varies from urban to rural areas[13],[14],[27].It has been found that in developing countries, attainment of education and having a paid job empower mothers to utilize maternal health services[28]. The survey done in Nepal demonstrated that sex of the child, household income, mother's education, partner's employment, and distance to health care provider all play various roles in determining health service utilization for children under age five[29].

From the fore-going, this study was undertaken to determine the utilization of maternal and child health care services and to identify factors which affect utilization among women of child bearing age in Southwest Nigeria. This will generate data for the sub-region and influence interventions and policies where necessary.

## Materials and methods

### Study area

Lagos State is located in the South-Western part of Nigeria. The State has a population of over 21million [30]and is made up of 16 urban and 4 rural Local Government Areas (LGAs). Oshodi-Isolo is one of the urban LGAs located in the North East of the State. It comprises 7 wards. According to 2006 population census, the total population of Oshodi-isolo was put at 621,509. The 2015 projected population is well over a million (1,506,399).The MMR of Oshodi-Isolo LGA is 443 per 100,000 [31]. The inhabitants are of varying educational, religious, occupational and ethnic backgrounds. The area is both residential and commercial. Formal and informal health care services are available in the study area. There are primary health care (PHC) centres, health posts, private clinics, pharmacies, patent medical stores, as well as complementary and alternative sources of services such as Traditional Birth Attendants (TBA), herbalist and spiritual homes. Many residents reside within five kilometres from formal health facilities that provide maternal and child healthcare services.

### Sample size and sampling procedure

The study was a community based, descriptive cross sectional study among women of child bearing age (15-49 years). Using the Cochran formula for calculating sample size for descriptive studies in populations>10, 000 (n = z ^2^pq/d^2^), an initial minimum sample size of 344 was calculated based on expected five percent error margin, 95% confidence interval and estimated proportion in the population who have particular characteristic of interest) of 0.65 from a previous similar study. ^15^However, to compensate for improperly filled questionnaires; the calculated sample size was increased by 10% to get the final sample size of 382 for the study.

Multistage sampling method was used to select the respondents. The first stage involved selection of two wards from the seven wards in Oshodi/Isolo by simple random sampling (Afariogun and Igbehinadun wards). The second stage was the selection of streets from the list of all the streets in the two selected wards. There were between 20-30 streets in Afariogun and Igbehinadun wardsand 10 streets were selected from each of them by simple random sampling. The third stage involved selection of houses in the selected streets using systematic sampling method. The average number of houses on each street is 40. The calculated sampling interval was two. The starting point on each of the streets was chosen by simple random sampling method and every second house was subsequently selected. The fourth stage involved selection of the eligible household from the houses using simple random sampling. One household in every house was selected. The last stage involved selection of respondents by simple random sampling (one eligible woman in each household) until the desired sample size was met.

### Data collection and quality checking

Data was collected using a structured, pretested, interviewer-administered questionnaire. Each interview lasted for about 20minutes and the data collection process lasted for one month. The questionnaire was designed to elicit information on socio demographic characteristics, utilization of maternal health care services (ANC, delivery, postnatal services and family planning) and utilization of neonatal and child health care services (immunization, growth monitoring, treatment of illness and nutritional services). Thirty questionnaires were pretested in Alimosho, another urban LGA in Lagos State and amended as appropriate. The interviewers were five trained voluntary community health extension workers (CHEWs) working in one the health centres in the Osodi-Isolo LGA. The interviews were conducted in English and Yoruba languages. The interviewers were frequently supervised on the field by the principal researcher to monitor data collection and provide necessary feedback. Data were entered manually into the Excel software twice by one of the co-researchers and a data entry manager, then exported to EPI info version 3.5.1 for analysis. Descriptive statistics such as frequencies and proportions were computed to describe the study population and variables. Inferential statistics (Chi square test) was done and level of significance was set at five percent (5%).

### Ethical consideration

Ethical approval was obtained from the Health Research and Ethics Committee (HREC) of Lagos University Teaching Hospital (LUTH) and assigned number ADM/DCS/HREC/APP/228. Appropriate community entry was done through the community leaders. Participation in the study was on a voluntary basis and informed written consent was obtained before the interview. Confidentiality of the information was assured and maintained by using an anonymous questionnaire.

## Results

A total of 382 questionnaires were administered but 371 were eventually valid for analysis. The study showed that 41.5% of the respondents were in the age group of 26–35 years with a mean age of 29±5years. Majority (92.5%) of the women were married and of Yoruba tribe (60.4%). A little above one third of them had two children. Seventy percent of them reside at a distance of ≤20 minutes travel time from the nearest health facility that renders maternal and child healthcare services. Only one quarter (25.3%) were educated beyond the secondary school level. Majority (80.6%) were employed with about 37.4% earning less than N20, 000 (66 US Dollars) monthly. (Table 1). Large proportions (60.9%) of the respondents’ spouses were between 31 to 40 years, many of whom had post secondary school education (44.2%). About 90.6% of them were gainfully employed.

**Table 1:** Socio demographic characteristics of respondents.

Variable (N = 371)	Frequency(n)	Percentages(%)
**Age (years)**		
≤20	13	3.5
21–25	78	21.0
26–30	154	41.5
31–35	86	23.2
36–40	32	8.6
41–45	8	2.2
**Mean age± SD: 29.23±5.178**		
**Marital status**		
Single	9	2.4
Married/Co-habiting	343	92.5
Widowed/Separated/Divorced	19	5.1
**Religion**		
Christian	195	52.6
Muslim	175	47.2
Traditional	1	0.3
**Parity**		
1	81	21.8
2	125	33.7
3	96	25.9
4	51	13.7
≥5	18	4.8
**Number of living children**		
1	92	24.8
2	145	39.1
3	90	24.3
4	32	8.6
≥5	12	3.2
**Mean number of children± SD**	**2.30±1.007**	
**Median**	**2**	
**Time to nearest health facility**		
≤10min	145	39.1
11-20min	122	32.9
21-30min	64	17.3
>30min	40	10.8
**Mean travel time ±SD**	**1.08±0.999**	
**Median**	**2**	

### Maternal health care utilization

Majority of the respondents received ANC in their last pregnancy from the health facilities (74.3%), out of which 65.6% did so in public health facilities. For those who had ANC at health facilities, approximately 48% registered in their second trimester and two thirds (65.9%) of the women visited the clinic four or more times while the remaining one third (34.1%) visited the clinic less than four times(Table 2).

**Table 2:** Utilization of maternal health care services among respondents.

Variables	Frequency(n)	Percentage (%)
**Place of antenatal care for last pregnancy**		
Health facilities only	210	56.6
TBA only	63	17.0
Health facilities and TBA	66	17.8
Did not go for ANC	27	7.3
Others	5	1.3
**Total**	**371**	**100.0**
**Types of health facility**		
Public health facility	181	65.6
Private health facility	95	34.4
**Total**	**276**	**100.0**
**Time of registration for ANC at health facility**		
1^st^ trimester (1-3months)	108	39.1
2^nd^ trimester (4-6months)	132	47.8
3^rd^ trimester (7-9months)	36	13.0
**Total**	**276**	**100.0**
**Number of antenatal care visits in last pregnancy**		
< 4 times	94	34.1
≥ 4 times	182	65.9
**Place of delivery**		
Public health facilities	149	40.2
Private health facilities	73	19.7
Traditional birth attendant	99	26.7
Home	28	7.5
Church/Mosque	18	4.9
Others	4	1.1
**Total**	**371**	**100.0**
**Birth attendant for deliveries for non institutional deliveries**		
Doctor	2	1.3
Nurse	22	14.8
TBA	102	68.5
Mother/mother-in-law	4	2.7
Other Relative	1	.7
Neighbour/friend	10	6.7
Older woman	8	5.4
**Total**	**149**	**100.0**
Reasons for choice of place of delivery*		
Nearness to home or work	128	34.5
Quality of care	139	37.5
Cost of services	102	27.5
Better attitude of health workers	92	24.8
Husband’s preference	79	21.3
Others	40	10.8

*Multiple responses

Most (59.7%) of the women had institutional deliverywhile 26.7% delivered at TBA centres and 7.5% at home. Out of the 95 respondents who did not deliver at health facilities, majority (68.5%) were delivered by TBAs while 14.4% were delivered by nurses.The quality of care(37.5%), nearness to home or work place(34.5%) and cost of services (27.5%) were the most frequent reasons for choice of place of delivery (Table 2).

Seventy eight percent utilized PNC in the last confinement. Majority (58.8%) made only one PNC visit after delivery and about half of them went in the 5^th^ or 6^th^week (50.2%). Most (56.8%) of the respondents who delivered at the health facilities were discharged at/after 24 hours post delivery. Newborn check up (98.6%), counselling on breastfeeding (92.0%), physical examination (74.4%), and family planning (73.7%) were the most frequently utilized services.

### Reasons for non attendance of antenatal and postnatal care

Only few (7.3%) did not receive ANC. Financial constraint (37%) was the major reason given, followed by the fact that they felt that they did not need to go since they were not sick (33%).The major reasons for not attending postnatal care were no reason (mother and child are healthy, so no reason for PNC) (61.0%), ignorance (19.5%) and long distance (11%). (Table 3)

**Table 3:** Reasons for non attendance antenatal and postnatal care.

Variable	Frequency(n)	Percentage
**ANC (N = 27)**		
No money	10	37.0
The clinic was too far	5	18.5
My husband did not allow	2	7.4
Health workers attitude	1	3.7
Did not need to	9	33.3
**Total**	**27**	**100.0**
**PNC (N = 82)**		
No time	4	4.9
Long distance	9	11.0
Don’t know about it	16	19.5
Others	3	3.7
No reason	50	61.0
**Total**	**82**	**100.0**

Majority (71.2%) of the respondents were not currently on modern family planning method. For the 28.8% who were currently using modern family planning; IUCD (32.7%), injections (24.3%) and implants (22.4%) were the commonly used ones. The decision to use was made by the respondents mostly (48.6%) and jointly by the respondents and their spouses in 36% of the cases. For those that did not use modern family planning method, the most frequent reason given was fear of side effects (41.7%).

### Utilization of child health care services

Almost all (98%) the respondents’ breastfed their babies, 52.9% of them commenced within 30 minutes postpartum. The proportion of respondents who practiced exclusive breastfeeding (EBF) was 31.5%. The mean duration for EBF was 5.57±1.125 months. Eighty six percent of the children had previously suffered diarrhoea since birth and the last episode was treated using ORS (82.4%), Zinc tablet (46.4%) and antibiotics (40.4%).

Majority (95.1%) of the respondents have utilized the health facilities for immunization services, growth monitoring (77.4%), nutritional counselling (64.7%) and treatment of illness (49.6%). Most of the respondents (96.6%) reported that their children were fully immunized for age but on sighting the immunization cards, this proportion dropped to 81%, and so only 19% were not immunized. Seventy eight percent utilized the growth chart for growth monitoring but majority (65.2%) reported that the progress of the child was not discussed by the health worker after recording the weight on the chart (Table 4)

**Table 4:** Utilization of child health care services among respondents.

Variable	Frequency(n)	Percentage (%)
Services utilized for child at the health facility*		
Immunization	353	95.1
Growth monitoring	287	77.4
Treatment of illness	184	49.6
Nutritional counselling	240	64.7
I have never taken my child to health facility	7	1.9
Others	22	5.9
**Child fully immunized for age, confirming from Immunization card**		
Yes	286	81.0
No	67	19.0
**Total**	**353**	**100.0**

*Multiple responses

### Factors associated with maternal and child health care services utilization

Higher maternal educational level and employment status of the women significantly increased utilization of ANC in health facilities. Also paternal educational level and employment status significantly increased utilization of ANC at the health facility (p = 0.016, <0.001). There was a significant association between age and use of delivery services at the health facility (p = 0.027). Other factors that also significantly influenced utilization of delivery services at health facilities were marital status(least among single mothers), maternal educational level and employment status. A statistically significant higher proportion of respondents whose spouses were employed and had at least secondary education utilized institutional delivery (p ≤0.001). (Table 5)

**Table 5:** Factors affecting respondents’ utilization of maternal health care services.

Variable	Health facility			X^2^	Pvalue
	Yes n(%)	No n(%)	Total N = 344		
FOR ANC					
**Age (years)**					
16–25	56(70.0)	24(30.0)	**80**(100.0)	10.78	0.375
26–35	186(82.7)	39(17.3)	**225**(100.0)		
36–45	34(87.2)	5(12.8)	**39**(100.0)		
**Total**	**276(74.3)**	**68(18.3)**			
**Marital Status**					
Married	259(80.9)	61(19.1)	**320**(100.0)	5.81	0.214
Not married	17(70.8)	7(29.2)	**24**(100.0)		
**Total**	**276(74.3)**	**68(18.3)**			
**Number of living children**					
1–2	172(78.0)	49(22.0)	**221**(100)	5.42	0.924
3–4	96(85.0)	17(15.0)	**113**(100)		
≥5	8(80.0)	2(20)	**10**(100)		
**Total**	**276(74.3)**	**68(18.3)**			
**Educational Status**					
No formal education	7(41.2)	10(58.8)	**17**(100)	43.32	<0.001*
Primary	22(57.9)	16(42.1)	**38**(100)		
Secondary	159(81.1)	37(18.9)	**196**(100)		
Post Secondary	88(94.6)	5(5.4)	**93**(100)		
**Total**	**276(74.3)**	**68(18.3)**			
**Employment Status**					
Employed	232(83.2)	47(16.8)	**279**(100)	11.31	0.004*
Unemployed	44(67.7)	21(32.3)	**65**(100)		
**Total**	**276(74.3)**	**68(18.3)**			
**FOR DELIVERY (N = 371)**					
**Age (years)**					
16–25	40(44.0)	51(56.0)	**91**(100)	12.64	0.027*
26–35	151(63.0)	89(37.0)	**240**(100)		
36–45	31(77.5)	9(22.5)	**40**(100)		
**Total**	**222(59.9)**	**149(40.1)**			
**Marital status**					
Married	213(62.1)	130(37.9)	**343**(100)	25.67	0.004*
Not married	9(32.1)	19(67.9)	**28**(100)		
**Total**	**222(59.9)**	**149(40.1)**			
**Numbers of living children**					
1–2	134(56.5)	103(43.5)	**137**(100)	37.08	0.175
3–4	80(65.4)	42(34.6)	**122**(100)		
≥5	8(66.7)	4(33.3)	**12**(100)		
**Total**	**222(59.9)**	**149(40.1)**			
**Educational status**					
No formal education	6(26.1)	17(73.9)	**23**(100)	68.71	<0.001*
Primary	13(29.5)	31(70.5)	**44(100)**		
Secondary	127(60.5)	83(39,5)	**210(100)**		
Post Secondary	76(80.9)	18(19.1)	**94(100)**		
**Total**	**222(59.9)**	**149(40.1)**			

* Significant

There was a significant association between utilization of PNC and number of ANC visits. Women who had minimum of 4 ANC visits (p = 0.007) and women who were discharged at least 24 hours after delivery in a health facility (p = 0.028) utilized PNC more. The highest proportions of modern family planning method userswere the older, higher educated women and those with at least three to four living children.

Utilization of both immunization and growth monitoring services increased as maternal age increased with a statistically significant difference (p = 0.061, 0.008). Also, there was a significant association with maternal education (p.0.002, <0.0010). Older and better educated women utilized the health facilities for treatment of illness for their children more than the younger and less educated ones and the association was significant (p = 0.007, <0.001).(Table 6)

**Table 6:** Associations between socio demographic data and child health care services utilization.

Variable	Immunization			X^2^	P	Growth monitoring			X^2^	P
	Yes N(%)	No n(%)	Total = 371			Yes n(%)	No n(%)	Total N = 371		
**Age**										
16–25	85(93.4)	6(6.6)	**91(100)**	10.52	0.061	61(67.0)	30(33.0)	**91(100)**	15.51	0.008*
26–35	229(95.4)	11(4.6)	**240(100)**			200(83.3)	40(6.7)	**240(100)**		
36–45	39(97.5)	1(2.5)	**40(100)**			26(65.0)	14(35.0)	**40(100)**		
**Total**	**353(95.1)**	**18(4.9)**				**287(77.4)**	**84(22.6)**			
**Number of living children**										
1–2	229(96.6)	8(3.4)	**237(100)**	5.22	0.516	187(78.9	50(21.1)	**237(100)**	8.91	0.178
3–4	113(92.6)	9(7.4)	**122(100)**			92(75.4)	30(24.6)	**122(100)**		
≥5	11(91.7)	1(8.3)	**12(100)**			8(66.7)	4(33.3)	**12(100)**		
**Total**	**353(95.1)**	**18(4.9)**				**287(77.4)**	**84(22.6)**			
**Educational status**										
Non formal	21(91.3)	2(8.7)	**23(100)**	14.79	0.002*	15(65.2)	8(34.8)	**23(100)**	28.22	<0.001*
Primary	38(86.4)	6(13.6)	**44(100)**			22(50.0)	22(50.0)	**44(100)**		
Secondary	207(98.6)	3(1.4)	**210(100)**			178(84.8)	32(15.2)	**210(100)**		
Post secondary	87(92.6)	7(7.4)	**94(100)**			72(76.6)	22(23.4)	**94(1000**		
**Total**	**353(95.1)**	**18(4.9)**				**287(77.4)**	**84(22.6)**			
**Variables**	**Treatment of illness**					**Nutritional services**				
Age										
**16–25**	30(33.0)	61(67.0)	**91(100)**	16.29	0.006*	56(61.5)	35(38.5)	**91(100)**	6.17	0.290
**26–35**	127(52.7)	113(47.3)	**240(100)**			155(62.0)	85(38.0)	**240(100)**		
										
**36–45**	26 (65.0)	14(35.0)	**40(100)**			29(72.5)	11(27.5)	**40(100)**		
Total	**183(49.6)**	**188(50.4)**				**240(64.7)**	**131(35.3**			
Number of living children										
**1–2**	106(44.7)	131(55.3)	**237(100)**	8.26	0.219	151(63.7)	86(36.3)	**237(100)**	5.91	0.423
**3–4**	72(57.7)	52(42.3)	**124(100)**			81(66.4)	41(33.6)	**122(100)**		
≥5	7(58.3)	5(41.6)	**12(100)**			8(66.7)	4(33.3)	**12(100)**		
Total Educational status	**188(49.4)**	**188(50.4)**				**240 (64.7)**	**131(35.3)**			
**Non formal**	11(47.8)	12(52.2)	**23(100)**	19.18	<0.001*	10(43.5)	13(56.5)	**23(100)**	8.97	0.029*
**Primary**	21(47.7)	23(52.3)	**44(100)**			23(52.3)	21(27.7)	**44(100)**		
**Secondary**	87(41.4)	123(58.6)	**210(100)**			143(68.1)	67(31.9)	**210(100)**		
**Post secondary**	84(68.1)	30(31.9)	**94(100)**			64(68.1)	30(31.9)	**94(100)**		
Total	**183(49.4)**	**188(50.4)**				**240(64.7)**	**131(35.3**			

* Significant

Respondents who received ANC at health facilities, delivered there and utilized PNC services were more likely to utilize the child health services. A higher proportion of respondents who received ANC at health facilities, and those who delivered at health facilities utilized both growth monitoring and curative services. These associations were statistically significant. Utilization of PNC was not significantly influenced by growth monitoring and curative healthcare service utilization though there was better use of these services among PNC users.

The multivariate analysis identified maternal schooling beyond the primary school level and spouse employmentas significant and independent predictors for the use of antenatal care services. Using ‘no formal education’ as a reference category, respondents with secondary education were six times more likely to use formal ANC (OR = 6.02, 95%CI 1.96–18.41). Similarly, those with post-secondary education were 32 times more likely to use formal ANC than those who had no formal education (OR = 32.87, 95%CI 7.91–136.45).

The predictors of skilled delivery services utilization were respondents and spouse educational status. Respondents with secondary education were four times more likely to use skilled delivery services (OR = 3.68, 95%CI1.18–11.46). Similarly, those with post-secondary education were ten times more likely to use skilled delivery services than those who had no formal education (OR = 9.62, 95%CI 2.69–34.42). (Table 7)

**Table 7:** Multiple logistic regressions (Multivariable analysis) of use of formal ANC & skilled delivery on the socio-demographic factors.

Variable	Odds Ratio	95% C.I		S. E.	Z	P
		Lower	Upper			
Use of ANC						
**Education status**						
Post Secondary/No formal	32.8725	7.9194	136.4500	0.7262	4.8095	0.0000*
Secondary/No formal	6.0177	1.9661	18.4185	0.5707	3.1445	0.0017*
Primary/No formal	2.2561	0.6903	7.3737	0.6042	1.3466	0.1781
**Employment status**						
Unemployed/Employed	1.3182	0.6402	2.7143	0.3685	0.7498	0.4534
**Marital**						
Married/Divorced/Separated/ Widowed	1.0486	0.3326	3.3054	0.5858	0.0810	0.9355
Single/Divorced/Separated/ Widowed	0.5109	0.0834	3.1302	0.9249	-0.7261	0.4677
**Spouse Education status**						
Post Secondary/No formal	1.0198	0.2764	3.7632	0.6661	0.0295	0.9765
Secondary/No formal	1.2892	0.3662	4.5389	0.6422	0.3956	0.6924
Primary/No formal	0.5190	0.1027	2.6242	0.8268	-0.7931	0.4277
**Spouse Employment status**						
Unemployed/Employed	0.2544	0.1080	0.5991	0.4371	-3.1319	0.0017*
CONSTANT	*	*	*	0.9057	-0.8128	0.4163
**Health facility for delivery**						
**Education**						
Post Secondary/No formal	9.6213	2.6891	34.4234	0.6504	3.4809	0.0005*
Secondary/No formal	3.6793	1.1814	11.4582	0.5796	2.2476	0.0246*
Primary/No formal	1.2470	0.3607	4.3113	0.6329	0.3487	0.7273
**Employment**						
Unemployed/Employed	1.1430	0.5981	2.1845	0.3305	0.4046	0.6858
**Marital**						
Married/Divorced/Separated/ Widowed	2.2715	0.8184	6.3050	0.5209	1.5751	0.1152
Single/Divorced/Separated/ Widowed	0.3593	0.0338	3.8167	1.2056	-0.8490	0.3959
**Spouse Educational status**						
Post Secondary/No formal	0.9541	0.2769	3.2875	0.6312	-0.0745	0.9406
Secondary/No formal	0.7479	0.2245	2.4911	0.6139	-0.4732	0.6361
Primary/No formal	0.1346	0.0203	0.8940	0.9661	-2.0760	0.0379*
**Spouse Employment status**						
Unemployed/Employed	0.6343	0.2637	1.5256	0.4478	-1.0167	0.3093
CONSTANT	*	*	*	0.8541	-1.6167	0.1060

*significant

The multivariate analysis identified women’s education as significant and independent predictor for the use of child health care services. Using ‘no formal education’ as a reference category, respondents with secondary education were seven times more likely to utilized immunization services (OR = 6.57, 95%CI 1.03–41.56) than those who had no formal education (Table 8)

**Table 8:** Multiple logistic regressions (Multivariable analysis) of utilization of child health care services on the socio-demographic factor (Maternal education).

Variable	Odds Ratio	95% C.I.		S. E.	Z	P
		Lower	Upper			
**Immnization**						
Post Secondary/No formal	1.1837	0.2291	6.1150	0.8378	0.2013	0.8405
Secondary/No formal	6.5714	1.0388	41.5695	0.9412	2.0004	0.0455*
Primary/No formal	0.6032	0.1117	3.2581	0.8606	-0.5875	0.5569
CONSTANT	*	*	*	0.7400	3.1775	0.0015
**Growth Monitoring**						
Post Secondary/No formal	1.7455	0.6538	4.6598	0.5010	1.1118	0.2662
Secondary/No formal	3.0624	1.1974	7.8320	0.4791	2.3360	0.0195*
Primary/No formal	0.5333	0.1882	1.5117	0.5316	-1.1825	0.2370
CONSTANT	*	*	*	0.4378	1.4358	0.1510
**Treatment of illness**						
Post Secondary/No formal	2.3273	0.9220	5.8747	0.4724	1.7880	0.0738
Secondary/No formal	0.7566	0.3192	1.7936	0.4404	-0.6334	0.5265
Primary/No formal	0.9960	0.3629	2.7337	0.5151	-0.0077	0.9939
CONSTANT	*	*	*	0.4174	-0.2084	0.8349
**Nutritional Counselling**						
Post Secondary/No formal	2.8690	1.1276	7.2996	0.4765	2.2120	0.0270*
Secondary/No formal	2.7147	1.1333	6.5031	0.4457	2.2406	0.0251*
Primary/No formal	1.4238	0.5162	3.9275	0.5177	0.6825	0.4949
CONSTANT	*	*	*	0.4206	-0.6238	0.5328

*significant

## Discussion

ANC saves the lives of mothers and babies by promoting and instituting good health before delivery and during the early postnatal period. This study found that the utilization of ANC was generally high. This proportion is higher than that reported in NDHS 2013(61%) [15]^1^ but lower than the proportion reported in India (99%), Kenya(89%), South east Nigeria (97%) [12],[32–33]. It is almost similar to figures reported in Ibadan, another city in southwest Nigeria where utilization was 78% [34]. This result may be attributed to the high proportion of women who had formal education and the fact that the study was carried out in an urban community where there is easy access to mass media. The majority (74.3%) that utilized ANC services did so at public health facilities which are in line with practices in Ilorin; North central Nigeria [35]. The reason for this is not farfetched as ANC services are usually offered at a subsidized rate in public health facilities. This may account for the long waiting time and poor attitude of health workers recorded in some studies as a reason for non utilization of maternal and child health services, as this preference will stretch the public facilities with negative consequence for utilization [26],[32].

The educational and employment status of respondents and their spouses were significant factors influencing the utilization of ANC services at health facilities. This finding was consistent with figures from our NDHS and other parts of the country [15],[26],[33]. This might be due to the fact that educated couples are better informed to make informed choices. Education provides a woman with a unique opportunity to improve her own health and that of her children. From the results, the predictive effect of higher levels of education on ANC utilization was pronounced, thus emphasizing the importance of formal education for the girl child.

There was a higher institutional delivery rate than reports from other studies done in Kenya (47%), Ethiopia (32%), Nigeria (Kaduna Northwest) (28%) and NDHS 2013 (30%) [10],[14].[15],[33].^1^About 40% of the respondents did not have institutional delivery, a proportion, which is a bit higher when compared with other parts of Nigeria [5],[14]. A study done in Ife Southwest Nigeria, reported non-facility delivery rate of 24% among pregnant women, while 70% home delivery rate has been reported in Northern Nigeria [25],[36]. Out of the 40% who delivered outside the health facility, only very few were delivered by skilled birth attendants. High morbidity and mortality is associated with unskilled delivery [37]. Similar to reports from another study [37], perceived quality of service was the most important factor which influenced the choice of institutional delivery.

The significance of maternal age in the utilization of institutional delivery has also been observed by other authors [10],[24],[33]. The youngest age group were the lowest proportion of users. Older women probably through exposures to health education during previous pregnancies were likely to make better choices. The younger women could be naive to take instructions and opinions from significant influencers like mothers and mothers in law especially in situations of low male involvement. It might also be because they are often less socio-economically empowered and as such, prefer facilities providing care at minimal cost. On further analysis, this apparent effect of age was downplayed.

Marital status was also a significant factor with the unmarried women having the lowest proportion of users while the married, educated and employed women have the highest proportion. The stigma and vulnerability associated with pregnancy outside wedlock were likely to deter unmarried respondents from having their babies in the health facility. Financial incapacity and lack of social support for these women with unwanted pregnancy have also been implicated [38–39]. The role of poverty from unemployment is also evident in this study by their significantly lower utilization of institutional delivery. The study has shown the effect of male educational and financial empowerment on institutional delivery. This is particularly important in the African setting as the male is usually the bread winner and major decision maker in health. There was an increased utilization with increasing number of children. This is consistent with report of a similar study done in southeast Nigeria [33]. It may be that these women experienced obstetric complications thereby prompting them to choose institutional delivery.

Of the 371 respondents, 78% received post natal check-up at least once during the six weeks following their last confinement. The utilization of PNC services was slightly higher than that found in Gondar Zuria district, Ethiopia (66.8%), and Lagos (66.2%) but much higher than the 35.3% found in Kaduna Northwest Nigeria and<10% reported in Anambra, Southeast Nigeria[17],[33],[38],[40–41]. The urban nature of the study may explain the high utilization of PNC, though for many, the actual visits still fall short of the minimum of four postnatal contacts recommended for all mothers and newborns by the WHO [42]. The significant association between socio-demographic characteristics and PNC service utilization is in line with previous studies [38],[40–41]. This further buttresses the importance of female education and empowerment. Other factors which increased PNC utilization were being discharged at least 24 hours after delivery and minimum of four ANC visits. These visits allow the women to receive information on positive maternal and child health seeking behaviour. With full implementation of the new WHO guideline on increased ANC visit [19], the PNC utilization is likely to improve, thus preventing more maternal and newborn morbidity and mortality.

According to the non-users of ANC, the major deterrent was financial, thus denying them the benefits of proper ANC. Mothers who did not utilize PNC saw no reason for it since mother and child appeared healthy. This reason has some socio-cultural backgrounds as it is a normal practice in many parts of the country for a new mother to be confined to the house for the first 40 days post-delivery unless for extremely important reasons.

One of the services received by respondents during PNC services in the last pregnancy was family planning counseling but surprisingly, this knowledge did not influence their family planning use as only about a third were current users at the time of study. This proportion is low though higher than the report of NDHS 2013, in which prevalence of modern method of contraceptive use was just 10% [15].A study conducted in Ethiopia showed that women who discussed about family planning issues with their husbands/partners were 10 times more likely to utilize family planning services than those women who do not discuss family planning issues [42]. Majority of non-users were afraid of side effects, or their spouses did not support family planning use. Family planning programmes need to address these issues through carefully designed messages and male involvement. The positive influences of number of living children and maternal education on the practice of family planning were revealed in this study. Some other authors had made similar observations [42–44].

Only about half of the respondents initiated breastfeeding early and even a lower proportion practiced exclusive breastfeeding. Though the proportion almost doubled the national average, it is far from satisfactory as the benefits of exclusive breastfeeding cannot be over-emphasized.

Diarrhoea affects child well-being and creates considerable demand for health services [45].WHO stated two simple and effective treatments for the clinical management of acute diarrhoea- the use of low concentration oral rehydration salts (ORS) and routine use of zinc supplementation [46]. Many of the women used ORS but the use of zinc was just 46.4%. The awareness of Zinc supplementation in diarrhoea appears to be low among them, this has negative implications for child survival.

Child health outcomes remain one of the most important parameters for measuring the overall social and economic well-being of a country. The higher utilization of childhood preventive healthcare services among respondents may be attributed to the minimal costs attached to these services especially in public health facilities, more than curative services. Many mothers were also likely practicing home treatment, hence the low facility consultation for illnesses. Other studies in urban Lagos also recorded higher utilization of preventive services [47–49].

Again the significance of maternal education was manifested in the utilization of child healthcare services. Children whose mothers are not educated are far less likely to be fully vaccinated than children whose mothers had more than secondary education (p = 0.002). They are also less likely to be taken regularly for growth monitoring (p<0.001) or for treatment of illness (p = 0.029). This is consistent with findings from Uganda, Nepal, NDHS 2013 and Lagos [11],[15],[24],[50],[51]. With an increase in the number of children, the rate of utilization dropped indicating that grand multiparous women were less likely to utilize health services for their children. Surprisingly, more of them had their babies in the health facility. This may be a case of ‘perceived expertise’ and brings to light, the importance of continuum of care and integration of maternal and child healthcare services. This will encourage good health-seeking behaviour for their children. It could also be financial as documented in other parts of Africa [52] inferring that household income and expenditure can predict utilization of child healthcare services. It was also shown that good maternal health-seeking behaviour (having at least four ANC visits) increased good child healthcare service utilization.

## Limitations of the study

The trained community health workers recruited as interviewers may have introduced some respondent-reporter bias.

The study was conducted in a sub-part of Lagos State and so results may not be generalized to the country.

A qualitative aspect could have been included to gain more insight into the subject.

## Conclusion

Though the utilization of some of the life lines (ANC, health facility delivery, PNC) by the respondents was high, lower rates were recorded for the utilization of family planning and the exclusive breastfeeding. Delivery at TBA centres was common. Only few women did not utilize any of the specified lifelines. Meanwhile utilization of preventive child health care services was better than curative services. Socio-demographic, socio economics factors and health-seeking behaviour were found to play significant roles in the utilization of maternal and child health services. The significance of maternal education in predicting utilization was very prominent in this study. These should be put into consideration in thedesign of appropriate interventions to further improve utilization. Further research, especially on the services with low utilization rates is needed to make the dream of the SDGs achievable.

## Recommendations

Programs to further enlighten women on the benefits of these life lines especially family planning and breastfeeding should be implemented. The well utilized maternal and child services like antenatal care and immunization can be used for this purpose. Furthermore the education of the girl child should be encouraged. Education enhances the economic status of mothers; it empowers them, increases the level of awareness, and builds independent decision making capacity in them. The inclusion of men as targets of family planning campaigns will have an important influence on its acceptance and usage. Interventions should focus on young women, those with low level of education and the poor. Spouses’ education and involvement should also be vital components of intervention programmes to improve utilization. A qualitative approach to this topic is also recommended in order to gain more insight.

## Supporting information

S1 File Abbreviations. (DOCX)

S2 File Questionnaire. (DOCX)

## References

[1] Schwind G, Broulet N, Hawes S. The lancet’s neonatal survival series.*The Lancet*.2005; 305(9474): 1845. 10.1016/S0140-6736(05)66607-X 15924975

[2] WHO, Unicef, UNFP, The world bank, The United Nations: Trend in Maternal Mortality 1990–2013.2013:1–56. http://www.who.Intl Reproductive Health. Last assessed 12/3/15.

[3] World Health Organization. Factsheet, Maternal Mortality: Fact sheet. Department of making pregnancy safer. 2008.http://www.who. Last accessed 23/4/15.

[4] UNICEF. The children_maternal_child health.2015. Error! Hyperlink reference not valid. Last accessed at 21/4/15.

[5] Iyaniwura CA and Yusuf Q.Utilization of Antenatal care and Delivery services in Sagamu, South Western Nigeria.*Afr J Reprod Health* 2009; 13[3]:111*–*122 20690266

[6] Tukur D, Oche MO. Determinants of antenatal care, institutional Delivery and postnatal care services utilization in Nigeria. Pan Afr Med J. 2015; 21: 321 10.11604/pamj.2015.21.321.6527 26587168 PMC4633744

[7] Saifuddin A, QingfengLi MA, Army OT.Maternal deaths averted by contraceptive use: an analysis of 172 countries. *The Lancet*. 2012; 308(9837):111–125. 10.1016/S0140-6736(12)60478-4 22784531

[8] World Health Organization. Maternal mortality, Fact Sheet 348. 2010. http://www.who.int/mediacentre/factsheets/fs348/en/index.html. Last accessed 23/4/15.

[9] WHO | Under-five mortality. 2016.www.who.int/gho/child_health_mortality/mortality_under_five/en/.Last assessed 18/6/16.

[10] Hailu D, Berhe H. Determinants of institutional childbirth service utilization among women of childbearing age in urban and rural areas of Tsegedie district Ethiopia.2014; 30(11): 1109–1117. 10.1016/j.midw.2014.03.009 24726608

[11] Mbonye KA. Prevalence of Childhood Illnesses and Care-Seeking Practices in Rural Uganda. *The Scientific World JOURNAL*.2003; 3:721–730. 10.1100/tsw.2003.52 12941972 PMC5974859

[12] Nzioki JM, Onyango RO, Ombaka JO. Socio-demographic factors influencing maternal and child health service utilization in Mwing: A rural semi arid district in Kenya. *American Journal of Public Health Reasearch*.2015; 3(1):21–30.

[13] Yarzever IS, Said IY. Knowledge and barriers in utilization of maternal health care services in Kano state, Northern Nigeria. *European Journal of Biology and Medical Science Research*.2013; 1(1):1–14.

[14] Idris SH, Sambo MN, Ibrahim MS. Barriers to utilization of maternal health services in a semi-urban community in northern Nigeria: *The clients' perspective*. *Niger Med J* 2013; 54:27–32 10.4103/0300-1652.108890 23661896 PMC3644741

[15] Nigeria Demographic and Health Survey. Preliminary report. National Population Commission NPC. 2013. http://dhsprogram,com/pub/pdf/PR41.pdf. Last accessed 4/6/15.

[16] Okafor IP, Bashir I, Dolapo DC. Maternal postnatal care utilization and associated factors: A community-based study among women on children bearing age in Lagos, Nigeria. *Journal of clinical sciences*.2013; 10(2):25–31.

[17] Tukai IU, Dlakwa HD, Bukar M, Audu BM, Kwayabura AS. Factors responsible for under utilization of postnatal care services in Maiduguri, north-eastern Nigeria. *Sahel Med J*. 2015:18:109–15.

[18] Zeleke D, Nega A, Gudina E. Maternal health care use among married women in Hossaina, Ethiopia.*BMC Health Serv Res*. 2015; 15: 365 10.1186/s12913-015-1047-1 26358062 PMC4566845

[19] World Health Organization (WHO).Antenatal care.2015. http://www.who.int/pmnch/…/aonsection111-2…Last accessed 23/6/15.

[20] World HO. Measles vaccination has saved an estimated 17.1 million lives. 2015. www.who.int/mediacentre/news/releases/2015/measles-vaccination/en. Last accessed 16/6/16.

[21] World Health Organization. Sustainable Development Goals (SDGs) 2016.http://www.who.int?sdg/en. Last accessed 20/2/17.

[22] World Health Organization. Infant and young child feeding 2016.http://who.int/mediacentre/factsheets/fs342/en. Last accessed20/5/16.

[23] Federal Ministry of Health.National Reproductive Health Policy and Strategy: Federal Ministry of Health. Academic journal 2012;10(2141):5897Available at: http://www.academicjournals.org/IJNM. Last accessed 23/4/15.

[24] Heidi WR, Emelta LW, Hendi T. Adolescent use of maternal and child health services in developing countries. *International family planning perspectives*.2006; 32(1): 2–10. 10.1363/3200606 16723297

[25] Ebuehi MO, Roberts AA, Inem V.Determinants of Utilization of Maternal Health Services aamong Traders in Lagos Markets. *NQJHM*.2006; 16 (2): 69–75

[26] Asekun-Olarinmoye EO, Bamidele JO, Egbewale BE, Oyebola I, Olarinmoye A, Ojofeitimi EO. Consumer assessment of perceived quality of antenatal care services in a tertiary health care institution in Osun State, Nigeria. *J Turk Ger Gynecol Assoc* 2009; 10:89–94.

[27] Okafor IP, Sekoni AO, Dolapo DC. Predictors of maternal health service utilization: A community based, rural urban comparison in Nigeria. *East African Journal of Public Health*.2014;11(1):620–630.

[28] Paudel M, Khanal V, Acharya B, Adhikari M.Determinants of Postnatal Service utilization in a Western District of Nepal: Community Based Cross Sectional Study. *J Women’s Health Care*.2013; 2:126.

[29] Chin B. Socio-Demographic Determinants of Child Health Services Utilization in Nepal.iHEA2007 6th World Congress: Explorations in Health Economics paper. 2007.

[30] Lagos Population 2016—World Population Review.2016. http://worldpopulationreview.com/world-cities/lagos-population/

[31] Lagos state ministry of health.Results of Comm. Based Survey (MMR) in Oshodi Isolo LG, Lagos.2016. www.lagosstateministryofhealth.com/…/results-of-comm-based-survey-mmr-in-oshodi.

[32] Sumithra S, Aswathy S, Sandeep S, Shobha P, Johnson A, Valsala L et al Maternal and Child health services utilization in married women of age 15–45 years.*J*. *Commun*. *Dis*.2006; 38 (1):102–105. 17370696

[33] Emelumadu OF, Ukegbu AU, Ezeama NN, Kanu OO, Ifeadike CO, Onyeonoro UU. Socio-demographic determinants of maternal health-care service utilization among rural women in Anambra State, South East Nigeria. *Ann Med Health Sci Res*. 2014; 4:374–82. 10.4103/2141-9248.133463 24971212 PMC4071737

[34] Dairo MD, Owoyokun KE.Factors affecting the utilization of antenatal care services inIbadan, Nigeria. 2010; 12(1):10–21

[35] Adewoye KR, Musa IO, Atoyebi OA, Babatunde OA. Knowledge and Utilization of Antenatal Care Services by Women of Child Bearing Age in Ilorin-East Local Government Area, North Central Nigeria. International *Journal of Science and Technology*. 2013:3(3); 17–22.

[36] Adamu YM, Salihu HM. Barriers to the use of antenatal and obstetric care services in rural Kano, *Nigeria*. *J Obstet Gynaecol* 2002; 22:600–3. 10.1080/0144361021000020349 12554244

[37] UNICEF. Delivery Care—UNICEF DATA 2016.http://data.unicef.org. Last assessed 18/6/16.

[38] Okafor IP, Bashir I, Dolapo DC. Maternal postnatal care utilization and associated factors: A community-based study among women on children bearing age in Lagos, *Nigeria*. *Journal of clinical sciences*.2013; 10(2):25–31.

[39] Yalem T, Tesfay G. Isabel G, Kerstin E, Hailemariam LM, San S. Determinants of antenatal and delivery care utilization in Tigray region, Ethiopia: a cross-sectional study. *International journal for Equity in Health*. 2013, 12:30 10.1186/1475-9276-12-30 23672203 PMC3658893

[40] Vishnu K, Mandira A, Rajendra K, Tania G. Factors associated with the utilization of postnatal care services among the mothers of Nepal: analysis of Nepal Demographic and Health Survey 2011. *BMC Women's Health* 2014, 14:19 10.1186/1472-6874-14-19 24484933 PMC3911793

[41] Tesfahun F, Worku W, Mazengiya F, Kifle M. Knowledge, Perception and Utilization of Postnatal Care of Mothers in Gondar Zuria District, Ethiopia: A Cross-Sectional Study. *Matern Child Health J*.2014; 18(10); 2341–2351 10.1007/s10995-014-1474-3 24770953 PMC4220106

[42] Abebe G, Nigatu R. Family planning service utilization in Mojo town, Ethiopia: A population based study. *Journal of Medical and Applied Biosciences*.2014; 6(2):101.

[43] Paschal AA, Ayamba MA. Factors influencing the uptake of family planning services in the Talensi District, Ghana. *The Pan African Medical Journal*. 2015; 20:10 10.11604/pamj.2015.20.10.5301 25995807 PMC4430143

[44] Malalu PK, Koseki A, Too R, Amoo C. Determinant or use of modern family planning. A case of baringo north district,Kenya. *Science journal of public health*.2014; l 2(5):424–430.

[45] Agbolade MO, Dpeolu IO, Ajuwon AJ. Knowledge and use of oral rehydration therapy among mothers of under-five children in a Military Barrack in Ibadan, Nigeria. *Afr*.*J*.*Biomed*.*Res*.2015; 18:7–15.

[46] World Health Organization.Zinc supplementation in the management of diarrhoea.2011. www.who.Int/elena/titles/…/zinc_diarrhoe. Last accessed 13/5/15

[47] Okafor IP, Dolapo DC, Onigbogi MO, and Iloabuchi IG, “Rural-urban disparities in maternal immunization knowledge and childhood health-seeking behaviur in Nigeria: a mixed method study,” *African Health Sciences*.2014; 14(2):339–347. 10.4314/ahs.v14i2.8 25320582 PMC4196403

[48] Adeyinka D, Oladimeji O, Adeyinka F, Aimakhu C. Uptake of childhood immunization among mothers of U5 in southwest Nigeria. *The internet journal of epidemiology*.2008; 7(2):1–6.

[49] Sule SS, Olawuyi O, Afolabi OT, Onajole A, Ogunowo BE. Caregivers Knowledge and Utilization of Child Health Services in an Urban District of Lagos, Nigeria. WAJM. 2013; 32(3): 163–172. 24122680

[50] Mbonye KA. Prevalence of Childhood Illnesses and Care-Seeking Practices in Rural Uganda. *The Scientific World JOURNAL*.2003; 3:721–730. 10.1100/tsw.2003.52 12941972 PMC5974859

[51] Chin B. Socio-Demographic Determinants of Child Health Services Utilization in Nepal.iHEA2007 6th World Congress: Explorations in Health Economics paper. 2007.

[52] Negussie T, Chepngeno G. Determinants of health care seeking for childhood illnesses in Nairobi slums. *Tropical Medicine and International Health*. 2005; 10(3): 240–245. 10.1111/j.1365-3156.2004.01381.x 15730508

