# Supplementary material for: Ending preventable maternal and child deaths in western Nigeria: Do women utilize the life lines?
Source: PLoS One. 2017 May 18;12(5):e0176195. doi: 10.1371/journal.pone.0176195 (PMC5436634; doi:10.1371/journal.pone.0176195)
Supplement: S1 File — (DOCX) [file pone.0176195.s001.docx]

# S1 File: Abbreviations

ANC: Antenatal care

EBF: Exclusive breastfeeding

IMR: Infant mortality rate

IUCD Intra uterine contraceptive device

LGA: Local government area

MCHS: Maternal and child health

MDG: Millennium development goals

MMR: Maternal mortality rate

MNCH: Maternal newborn and child health

NMR: Neonatal mortality rate

NDHS: Nigeria demographic and health survey

OR: Odd ratio

PHC: Primary health center

PNC: Postnatal care

TBA: Traditional birth attendance

SBA: Skilled birth attendance

SDG: Sustainable developmental goals

SSA: Sub Sahara Africa

UNICEF: United nation children fund

U5MR: Under –five mortality rate

WHO: World health organization
