# Supplementary material for: Ending preventable maternal and child deaths in western Nigeria: Do women utilize the life lines?
Source: PLoS One. 2017 May 18;12(5):e0176195. doi: 10.1371/journal.pone.0176195 (PMC5436634; doi:10.1371/journal.pone.0176195)
Supplement: S2 File — (DOCX) [file pone.0176195.s002.docx]

**S2 File: Questionnaire**

**SECTION A: BIODATA**

Demographic information

1. Age as at last birthday

2. Religion: (a). Christian (b). Muslim (c). Traditional

3. Marital status: (a). Single (b). Married/Co-habiting

(c).Widowed/Separated/Divorced 4. Educational status: (a). No formal education (b). Primary (c).Secondary (d).Post secondary

5. Employment status: (a). Employed (b). Unemployed

6. Average monthly income (if employed): (a). < 20,000 (b).20, 000-30,000

(c).40, 000-50,000 (d).>50,000

7. Parity: ………….

8. Number of living children: …………

9. Time to nearest health facility: (a). <10min (b). 10-20min (c). 20-30min (d). >30min

**Spouse/partner demographic information**

10. Age of spouse/partner

11. Educational status: (a). No formal education (b). Primary (c). Secondary ( (d).Post secondary

12. Employment status: (a). Employed (b). Unemployed

**SECTION B: UTILIZATION OF MATERNAL HEALTH CARE SERVICES**

**Antenatal care (ANC)**

13. For your last pregnancy, where did you go for antenatal care?

(a) Health facilities only (b) TBA only (c) Health facilities and TBA

(d) Did not go for ANC (e) Others (Specify)

14. If health facilities. Is it (a) Public health facility (b) Private health facility?

15. If you did not go for antenatal care, why not?

(a). No money (b). The clinic was too far (c). My husband did not allow (d). Health workers attitude (e). I did not need to (f). Others (specify).

16. If you attend antenatal care at health facilities, when did you register?

(a). 1^st^ trimester (1-3months) (b). 2^nd^ trimester (4-6months)

(c). 3^rd^ trimester (7-9months).

17. How many times did you go for antenatal visit? (a).< 4 times (b). ≥ 4 times

| QUESTIONS | YES | NO |
| --- | --- | --- |
| 18. Did you do weight monitoring? |  |  |
| 19. Did you do blood pressure monitoring? |  |  |
| 20. Did you do general examination including abdominal examination ? |  |  |
| 21. Did you receive TT immunization? |  |  |
| 22. Did you take antimalaria prophylaxis (IPT)? |  |  |
| 23.Did you take iron tablet? |  |  |
| 24.Did you take folic tablet? |  |  |
| 25. Did you do blood investigations? |  |  |
| 26. Did you do urine investigations? |  |  |
| 27. Did you do counseling on HIV test? |  |  |
| 28. Did you receive education on signs of pregnancy complication? |  |  |

**Utilization of Delivery services**

29. Where did you deliver your baby?

(a). Public health facilities (b). Private health facilities (c) .Traditional birth attendant (d). Home (e). Church/Mosque (f). Others (Specify).

30. If not at health facilities, who delivered the baby?

(a). Doctor (b). Nurse (c).TBA (d). Mother/mother-in-law

(e). Other Relative (f). Neighbour /friend (g). Older woman (h).Others (specify)

31. What are your reasons for choice of place of delivery?( Tick all that apply)

(a). Nearness to home or work (b). Quality of care (c). Cost of services

(d). Better attitude of health workers (e).Husband preference (f). Others (Specify)

**Utilization of Postpartum/Postnatal care PNC services**

32. If you delivered at the health facility, when did you go back home after delivery?

(a). <24hr (b). ≥24hr

33. Did you go for post natal check up in your last pregnancy? (a). Yes (b). No

If No, go to question 37

34. If yes, when did you go for the check up?

(a). 1^ST^-2^ND^ week post natal (b). 3^RD^-4^TH^ week post natal (c). 6 weeks and after post natal

35.How many times do you go for postnatal visit? (a).1 (b). 2 (c). 3 (d). 4

36. Which of these services did you received at the clinic? (Tick all that apply)

(a). Physical Examination (b). Counselling on breastfeeding

(c). Family planning counselling (d).Newborn check-up (e). Others (specify)

37. If you did not go for postnatal check up, why not?(a). No time (b). Long distance (c). I don’t know about it (d). Others (specify)

(e). No reason

**Family planning services**

38. Are you currently using any modern family planning method? (a). Yes (b). No

If no, go to question 41

39. If yes, which one?

(a). IUCD (b). Pills (c). Injections (d). Implant (e). Others (specify)

40. Who took the decision to use the modern family planning?

(a). Self. (b). Spouse (c). Both (d). Others (Specify)

41. If you are not on any modern family planning method, why not?(Tick all that apply)

(a). Husband does not support it (b). Fear of side effect

(c).My religion does not permit it use (d). I am planning for another child

(e). Fear of inability to get pregnant when desired (f). Others (specify)

(g). No reason

**SECTION C**

**Utilization of newborn and child health care services**

42. Did you breastfeed your child? (a). Yes (b). No

If No, go to question 46.

43. If, Yes, did you initiate breastfeeding within 30minutes of delivery? (a).Yes (b).No

44. Did you practice exclusive breastfeeding? (a). Yes (b). No

45. If Yes, for how long?...................................... (Month)

46. Has your child ever had diarrhea? (a). Yes (b). No

47. IF yes, what did you do to treat it the last episode? (Tick all that apply)

(a). Antibiotics (b). ORS (C). Herbal concoction (d). Zinc tablet

(e). Others (specify)

48. Since the birth of your child, which of these services have you utilized at the health facility? (Tick all that apply)

(a). Immunization (b). Growth monitoring (c). Treatment of illness (d).Nutritional counseling (e). I have never taken my child to health facility (f).Others (specify)

49. If child was immunized, is the child fully immunized for age?(self report)

(a).Yes (b). No

50. Confirming with Immunization card, is the child fully immunized for age?

(a). Yes (b).No (c).card not available

51. If growth monitoring was one of your purposes for the visit, did you use the growth chart? (a)Yes or (b) No

52. If yes, did the health worker explain/discuss the progress of your child with you after recording the weight on the growth chart? (a).Yes (b).No
